# Supplementary material for: Mapping Histoplasma spp. in bats and cave ecosystems: evidence from midwestern Brazil
Source: Appl Environ Microbiol. 2025 Aug 12;91(9):e00335-25. doi: 10.1128/aem.00335-25 (PMC12442369; doi:10.1128/aem.00335-25)

Figure S1. qPCR amplification curves for the positive control strain CAO4 (*Histoplasma suramericanum*), using genomic DNA diluted across a range from 0.1 ng to 100 ng. The curves illustrate the fluorescence signal over successive PCR cycles, demonstrating the assay’s sensitivity and dynamic detection range across varying DNA input concentrations.


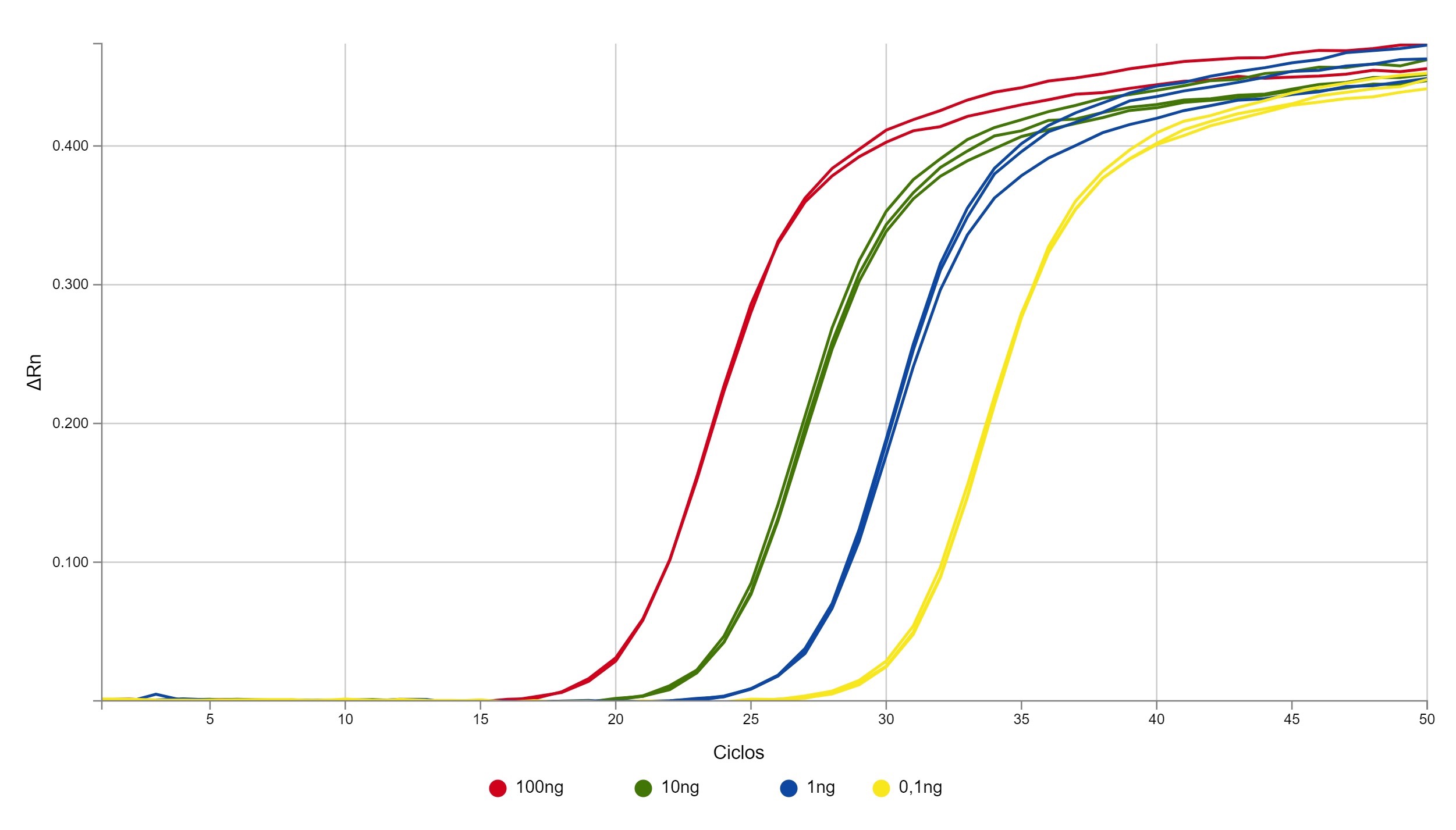

Supplement: Fig. S1 — qPCR amplification curves for the positive control strain CAO4 (Histoplasma suramericanum), using genomic DNA diluted across a range from 0.1 ng to 100 ng. [file aem.00335-25-s0001.docx]
